# Host Resistance and Behavior Determine Invasion Dynamics of a Detrimental Aquatic Disease

**DOI:** 10.1002/ece3.70393

**Published:** 2024-10-03

**Authors:** Mikko Koivu‐Jolma, Raine Kortet, Anssi Vainikka, Veijo Kaitala

**Affiliations:** ^1^ Department of Physics University of Helsinki Helsinki Finland; ^2^ Department of Environmental and Biological Sciences University of Eastern Finland Joensuu Finland; ^3^ Organismal and Evolutionary Biology Research Programme, Faculty of Biological and Environmental Sciences University of Helsinki Helsinki Finland

**Keywords:** *Aphanomyces astaci*, *Astacus astacus*, cannibalism, crayfish, crayfish plague, emergent disease, epidemiology, necrophagy, transmission

## Abstract

Understanding the role of variation in host resistance and the multitude of transmission modes of parasites infecting hosts with complex behavioral interactions is essential for the control of emerging diseases. We used a discrete stage model to study the invasion dynamics of crayfish plague—an example of a detrimental disease—into a naïve host population that displays within‐population variation in resistance of environmental infections and juvenile classes that are safe from contacts with adults. In the model, infection sources include four age classes of crayfish, contaminated carcasses, and free‐dwelling zoospores. Disease transmission occurs via environment with a threshold infection density and through contacts, cannibalism, and scavenging of disease‐killed conspecifics. Even if the infection is fatal, coexistence of the host and the parasite can be facilitated by variance of resistance and survival of the hiding juveniles. The model can be applied in the control of emerging diseases especially in crayfish‐like organisms.

## Graphical abstract

To control emerging diseases, we need to understand the effects of variation in host resistance and the multitude of transmission modes of parasites combined with hosts' complex behavioral interactions. Based on a discrete stage model, we propose that even if the infection is fatal, coexistence of the host and the parasite can be facilitated by variance in resistance and the partial separation of juveniles from adults.

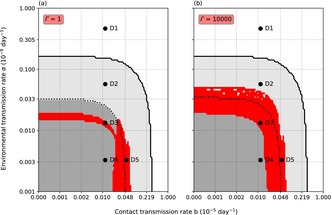

## 1 Introduction

Epidemiological models often assume uniform immunological response among susceptible hosts (Miller, White, and Boots 2005; Gilligan and van der Bosch 2008; Diekmann, Heesterbeekm, and Britton 2013), yet organisms display both intrinsic and acquired variation in parasite resistance and tolerance with ecological relevance (Barger 1989; Boots et al. 2014; Mazé‐Guilmo et al. 2014). Variation in host's capacity to eliminate disease agents (parasite resistance) can affect the impact and establishment of emerging diseases (Hulse et al. 2023). The development of general epidemiological models with multiple transmission routes is difficult since parasite transmission modes are often system‐specific, and their parameters can be poorly measurable (Antonovics 2017). Yet, understanding the multitude of transmission modes and their system‐specific qualities is required to yield ecologically feasible predictions for empirical research, conservation, and management of the disease‐impacted species and to control human diseases (Murray 2009; Woolhouse 2019). Disease transmission can occur via environment or through encounters between hosts. Correspondingly, contact functions are generally classified as density‐dependent or frequency‐dependent (Diekmann, Heesterbeekm, and Britton 2013; Keeling and Rohani 2008). However, the main modes of transmission alone cannot describe transmission cycles in fluctuating populations (Borremans et al. 2017) or in populations with behaviorally complex contact types (McCallum, Barlow, and Hone 2001). Similarly, assuming only the main type of contact in transmission can exclude major epidemiological dynamics (Uricchio et al. 2022).

Crayfish plague, an emergent disease caused by the oomycete *Aphanomyces astaci* of North American origin, has been deadly to the native European, Asian, Australian, and South American crayfish populations, including those of the noble crayfish (*Astacus astacus*; e.g., Jussila et al. 2014). Following the initial introduction, presumably via ballast water and later along with the non‐native crayfish hosts, this microparasite has spread widely on the Eurasian continent, reaching Turkey and even adjacent islands including Ireland (Holdich and Gherardi 1999; Jussila et al. 2014; Kokko et al. 2018; Mirimin et al. 2022). *A. astaci* produces infective zoospores in the cuticle of both living and dead crayfish. Despite some indications of decreased virulence in certain parasite strains and the possibility for latent infections (Jussila et al. 2021; Panteleit et al. 2018; Viljamaa‐Dirks et al. 2011), crayfish native to other regions than North America generally remain vulnerable to the disease (Becking et al. 2015; Martínéz‐Ríos et al. 2022; Mirimin et al. 2022; Svoboda et al. 2017). The strains infecting invasive signal crayfish (*Pasifastacus leniusculus*) and other coevolved hosts (Ungureanu et al. 2020; Mojžišová et al. 2020) remain particularly lethal to the hosts outside the disease's native range due to the conflicting selection pressures on parasite virulence between resistant native and vulnerable novel hosts (Becking et al. 2015; Jussila et al. 2013; Makkonen et al. 2014; Martínéz‐Ríos et al. 2022). Importantly, crayfish display variation in immune responses at individual and population levels (Dragičević et al. 2021; Gruber et al. 2014). This suggests that there is within‐population variation also in resistance against the disease, which might promote the survival of the most resistant individuals during an epidemic.

While not all details of transmission dynamics have been resolved empirically, environmental transmission through water is the key transmission route for *A. astaci* (Oidtmann et al. 2002; Svoboda et al. 2017). To cause the disease, a certain minimum number of zoospores must reach a susceptible host (Becking et al. 2015; Makkonen et al. 2014), leading to increased transmission with increased number of infected individuals. Similarly, high population density of the crayfish inherently increases the likelihood of contacts between the hosts increasing also contact‐dependent transmission. Although detailed research on contact transmission in *A. astaci* is lacking, locally increasing zoospore density through contacts and microscale chemotaxis of the zoospores toward the crayfish (Cerenius and Söderhäll 1984; Svensson 1978) may critically increase disease transmission (Koivu‐Jolma et al. 2023; Strand et al. 2012).

Crayfish display facultative scavenging, cannibalism, and intraspecific necrophagy (scavenging on dead conspecifics; Guan and Wiles 1998; Houghton, Wood, and Lambin 2017), which have been shown to increase dietary opportunities in resource‐poor environments (Brown and Norris 2004; Mastrantonio et al. 2021). To our knowledge, no experimental or observational research on the effect of cannibalism in disease dynamics specific to crayfish plague has been published. Theoretically, cannibalism can stabilize the host parasite dynamics by decreasing the total zoospore production (Koivu‐Jolma et al. 2023). Necrophagy of infected carcasses can create a significant transmission route for aquatic parasites during epidemics, although it may also speed up clearance of environmental infection sources (Hamano et al. 2015; Imhoff et al. 2012; Schönherz et al. 2012). Transmission of *A. astaci* through necrophagy has not been researched, but during necrophagy, a susceptible crayfish can spend prolonged time near infected carcasses at time when the zoospore production rates are at the highest (Makkonen et al. 2013). Thus, necrophagy might amplify epidemics by leaving the potentially surviving individuals exposed to infectious food sources.

Host life‐history affects epidemiological dynamics. Many crayfishes reproduce in discrete intervals (Abrahamsson 1971), leading to seasonal population size peaks that may affect the disease dynamics compared to systems with continuous reproduction (Borremans et al. 2017). Models with birth pulses have been used in the study of competitive population interactions (Chen and Zhao 2016; Jin, Zhien, and Maoan 2004; Jin, Maoan, and Guihua 2005; Ledder et al. 2021; Liu and Chen 2007; Nedorezov 2011; Sadykova et al. 2009), host–parasitoid interactions (Emerick and Singh 2016; Singh and Nisbet 2007), and crayfish population dynamics (Sadykova et al. 2009). Additionally, crayfish plague epidemiology may be complicated by the behavioral interactions between juveniles and adults, if the juveniles avoiding adults simultaneously avoid contact transmission of the disease (Olsson and Nyström 2009).

Here, we study how the invasion dynamics of an aquatic emerging disease in a model with four distinct infection routes. We use the noble crayfish as the basis for the host and highly virulent *A. astaci* strain for the parasite. In the model, crayfish reproduce in bouts that define the annual population dynamics. Within the year, the dynamics of the crayfish and crayfish plague follow ordinary differential equations describing continuous growth, death, and infections. In comparison with our earlier modeling of the crayfish plague epidemics (Koivu‐Jolma et al. 2023), we here introduce discrete reproduction pulses, constant variation in host resistance, and ability of juveniles to hide from adults.

We examine short timescale dynamics and focus on a 20‐year postinvasion period of epidemics. First, we focus on how the invasion depends on the rates of environmental and contact transmissions. Following this, we examine the host survival and the possibility of coexistence with respect to the original number of infected hosts introduced to the population. Finally, we study the effects of cannibalism, intraspecific necrophagy, and host resistance on the invasion dynamics.

We hypothesize that variation in host resistance can make the coexistence of the host and the disease possible at low crayfish densities.

## 2 Materials and Methods

### 2.1 Overview

We use a mixed‐time compartment model in which a discrete‐time submodel describes the dynamics from one year to another, including the development of the juvenile crayfish, maturation, and reproduction, and in which a continuous‐time submodel describes the interaction dynamics of the crayfish and crayfish plague within the discrete years. In this formulation, the host has an annual reproduction cycle and the parasite reproduces continuously. Within‐year rates include parasite transmission, natural mortality of the crayfish, and the decay and clearance of the parasite from the environment. Disease transmission through contacts, cannibalism, and intraspecific necrophagy are modeled as frequency‐dependent processes and infection through the environment as a density‐dependent process.

**FIGURE 1:**
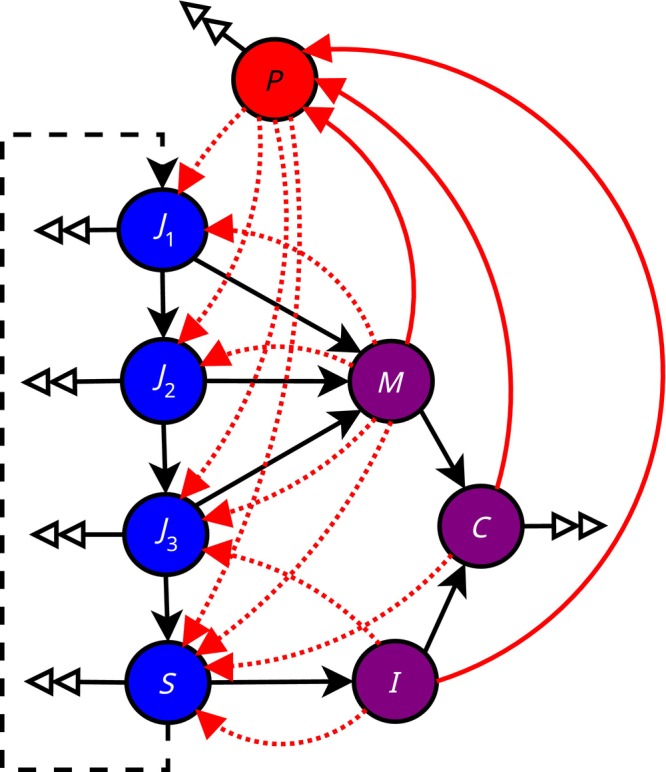
The crayfish plague model. The circles represent densities of the model classes. Blue circles represent the parasite free classes: *J*_1_–*J*_3_ are the juvenile classes from younger to older and *S* is the susceptible adult crayfish class. Purple circles represent the infected crayfish classes: *M* denotes the infected juvenile crayfish, *I* the infected adult crayfish and *C* the infected crayfish carcasses. The red circle *P* is the parasite zoospore class. Solid black lines describe the movement of individuals from one class to another, or in the case of double arrows, the complete removal from the system through death. Solid red lines indicate the addition of zoospores to *P* class. Dotted red arrows show the effect of the parasite. Dashed black line is the crayfish reproduction.

The model (Figure 1) has one oomycete zoospore compartment reflecting the density of free‐dwelling spores in the water (*P*), four susceptible crayfish age‐class compartments (*J*_1_, *J*_2_, *J*_3_, *S*), two infected crayfish compartments(*M*, *I*), and one infected carcass compartment (*C*).

We assume that the crayfish do not differentiate between parasite‐free and infected carcasses, and thus treat all crayfish compartments uniformly with respect to necrophagy. An infection threshold applies to the density‐dependent environmental transmission, and a contact is assumed to lead to an infection. Thus, regardless of the efficiency of digestive system as a transmission route, both cannibalism and necrophagy imply parasite transmission if the victim is infected, with the difference being only that in the former case, the population density is reduced without increase in total transmission when the cannibalized individual is uninfected.

For simplicity, we assume that hosts have no tolerance against the parasite (i.e., prolonged ability to transmit the disease without succumbing to it). The model was simplified by disregarding the effects of temperature and other environmental factors—including external disease vectors—that could affect the population dynamics or either the host or the parasite, or the transmission process. Moreover, the present model is a completely ecological such that trait composition in a population is constant over time.

### 2.2 The Model

We consider the invasion dynamics of a parasite with four transmission routes: transmission through (1) environment, (2) contact, (3) cannibalism, and (4) intraspecific necrophagy (Table 1). Because the infected crayfish usually succumb quickly to the disease, that is, females die before their brood is released, we excluded vertical transmission. The environmental transmission (1) represents the standard density‐dependent transmission that increases with the number of increasing infected hosts and leads nonlinearly to new infections when the threshold spore density is exceeded. The contact transmission (2) between alive crayfish increases not only with the proportion of infected individuals but also with increasing population density. The cannibalism‐associated transmission occurs as (2) but only in age‐dependent manner such that younger infected crayfish infect older (and bigger) crayfish. The intraspecific necrophagy is a form of cannibalism where individuals remove infected deceased conspecifics from the environment independently of their age (and size) but become infected themselves. As such, many of the transmission routes are parallel, and it is impossible to make clear predictions for their epidemiological outcomes without quantitative analysis of the model.

**TABLE 1:** The different transmission routes, their ranges, and their affected host classes.

Transmission route	Range	Infection threshold	1. and 2. year juveniles can transfer	3. year juveniles can transfer	Adults can transfer
Environment	Long	Yes	To all classes	To all classes	To all classes
Contact	Contact	No	To 1. and 2. year juveniles	To 3. year juveniles and adults	To 3. year juveniles and adults
Cannibalism	Contact	No	No	To adults	To adults
Intraspecific necrophagy	Contact	No	To adults	To adults	To adults

Each discrete time step (including 365 days of continuous‐time processes) begins with a reproductive bout, modeled as the season's initial condition for the first juvenile stage and with development of juveniles to the next age classes, as well as with the maturation of the third juvenile class (see the initial conditions).

The dynamics of parasite zoospore (*P*) density are continuous, and zoospores are produced continuously. The temporal dynamics of the crayfish plague can be followed by tracking the densities of infected juvenile and adult crayfish (*M*, *I*).

The full differential equation model of the crayfish infected by crayfish plague is described as follows:

(1) $$\frac{\mathrm{dP}}{\mathrm{dt}}=\mathrm{aM}+\mathrm{aI}+\mathrm{dC}-\gamma_{P}P$$

(2) $$\frac{dJ_{1}}{\mathrm{dt}}=\left(-\mu_{J}-\mathrm{\alpha z}\left(P\right)P-\mathrm{bM}\right)J_{1}$$

(3) $$\frac{dJ_{2}}{\mathrm{dt}}=\left(-\mu_{J}-\mathrm{\alpha z}\left(P\right)P-\mathrm{bM}\right)J_{2}$$

(4) $$\frac{dJ_{3}}{\mathrm{dt}}=\left(-\mu_{J}-\mathrm{cS}-\mathrm{\alpha z}\left(P\right)P-\mathrm{bI}-\mathrm{bM}\right)J_{3}$$

(5) $$\frac{\mathrm{dS}}{\mathrm{dt}}=\left(-\mu_{S}-\mathrm{\alpha z}\left(P\right)P-\mathrm{gC}-\mathrm{cM}-\mathrm{bI}-\mathrm{bM}\right)S$$

(6) $$\frac{\mathrm{dM}}{\mathrm{dt}}=\left(\mathrm{\alpha z}\left(P\right)P+\mathrm{bM}\right)\;\left(J_{1}+J_{2}+J_{3}\right)+\mathrm{bI}J_{3}-\left(\mathrm{cS}+\mu_{J}+\mu_{\inf}\right)M$$

(7) $$\frac{\mathrm{dI}}{\mathrm{dt}}=\left(\mathrm{\alpha z}\left(P\right)P+\mathrm{gC}+\mathrm{cM}+\mathrm{bI}+\mathrm{bM}\right)S-\left(\mu_{S}+\mu_{\inf}\right)I$$

(8) $$\frac{\mathrm{dC}}{\mathrm{dt}}=\mu_{\inf}\;\left(I+M\right)-\mathrm{gSC}+\mu_{S}I+\mu_{J}M-\left(\gamma_{T}+\gamma_{C}\right)C$$

During the annual cycle, let *T* = 365 denote the last time point of a year. The initial conditions for the next year are given as *P*(0) = *P*(*T*), *J*_1_(0) = *r*_S_ × *S*(0), where *r*_S_ is the per capita reproductive rate, *J*_2_(0) = *J*_1_(*T*), *J*_3_(0) = *J*_2_(*T*), *S*(0) = *S*(*T*) + *J*_3_(*T*), *M*(0) = *M*(*T*), *I*(0) = *I*(*T*), and *C*(0) = *C*(*T*).

Equation 1 describes the continuous release of the parasite zoospores into the environment by the alive (*M*, *I*) and deceased (*C*) infected crayfish at rates *a* and *d*, respectively. The zoospores decay in the environment at a constant rate *γ*_P_. In (Equation 2), juvenile crayfish, *J*_1_, are produced by the adult crayfish, *S*, at a rate *r*_S_ during each reproductive bout.

The juveniles in age classes *J*_1_ and *J*_2_ move to the next age class at the end of the year, and juveniles in the age class *J*_3_ mature to class *S*. The two smallest juvenile classes *J*_1_ and *J*_2_ were treated separately to comply with the ecologically relevant age structure used in the crayfish demographic references (e.g., Abrahamsson 1966). In this form, mortality rates could differ between juvenile classes, but for simplicity, we used uniform natural mortality (*μ*_J_ Equations (2), (3), (4)) in all juvenile classes. Further, the mixed‐time model together with the assumption of behavioral segregation between the juveniles and adults can cause potentially large and uneven oscillations in juvenile densities between years, in which the delays depend on the compartment structure. The mortality in the age class *J*_3_ is further increased by the cannibalism by the larger adult crayfish (*cSJ*_3_ Equation 4), whereas the adults face only the background mortality (*μ*_S_). We assume that cannibalism rate within the two youngest age classes is limited due to the sheltering in natural environment and part of the natural mortality rate that includes the predation by other species. Additionally, they are assumed safe from contact infections by the older classes due to behavioral isolation. Thus, there is no explicit cannibalism term for *J*_1_ and *J*_2_.

We also assume that due to the predation, the carcasses of the young individuals remain scarce, and behavioral host avoidance prevents the young age classes from consuming the carcasses of the older classes. Thus, the young individuals rely on environmental sources of food similarly to Koivu‐Jolma et al. (2023). All individuals are vulnerable to environmental infection by zoospores dispersing via water column (*α*z(*P*)*J*_1–3_, *αz*(*P*)*S* Equations (2), (3), (4), (5)). Infection by zoospores has an infection threshold denoted with *z*(*P*) (see below). The juveniles as well as the adults may also be infected due to direct contact with diseased individuals (*bIJ*_1–3_, *bMJ*_1–3_, *bSI*, *bSM* Equations (2), (3), (4), (5)). The adults may become infected by cannibalizing diseased juveniles (*cSM* Equation 5) and by intraspecific necrophagy (*gSC* Equation 5).

Infected individuals are moved to the classes of infected juveniles (*M* Equation 6) or adults (*I* Equation 7). The population size of infected juveniles, *M*, increases due to environmental and contact transmissions of the healthy juveniles and decreases due to cannibalism, natural mortality, and virulence of the parasite, where virulence parameter *μ*_inf_ describes the mortality rate due to the infection. The infected juveniles do not have time to age or mature due to the high mortality. Moreover, we assume that no recovery, tolerance, or immunization take place. Infected carcasses, *C*, increase with the virulence of the parasite (*μ*_inf_) and natural mortality (*μ*_J_, *μ*_S_) of the infected individuals, lose their infectiveness at rate *γ*_C_ (Equation 8), and decrease from being scavenged or through decay (*γ*_T_). The decay of the carcasses is due to the natural decomposition and scavenging by other scavenging species. Parameter *g* has a dual meaning: It denotes the scavenging rate of infected carcasses by the adult crayfish and the rate of transmission from scavenging an infected carcass.

The infection threshold by the zoospores is defined as a logistic function:

(9) $$z\left(P\right)=1/\left(1+\exp\;\left(-\frac{\left(P-∆\right)}{\Gamma }\right)\;\right)$$

The threshold value is denoted by *∆*. For high enough *∆* when *P* = 0, then *z*(*P*) ≈ 0. Respectively, when *P* >> *∆*, then *z*(*P*) ≈ 1. The logistic function (9) has a sigmoidal shape where an abrupt change from none to zoospore infections occurs around the value *P* = *∆* with *z*(*Δ*) = 1/2. Parameter *Γ* implicitly captures the individual variance of resistance to the infection—shown to significantly influence epidemiological dynamics (e.g., Barger 1989; Boots et al. 2014). Low/high values of *Γ* represent low/high variance of resistance. Thus, very low values of *Γ* represent clonal responses to the infection, and high values of *Γ* represent diverse immunological responses to the infection.

### 2.3 Model Calibration

We adapted the crayfish life‐history parameters (Table 2; Koivu‐Jolma et al. 2023) from Abrahamsson (1966, 1971) and the effects of infection parameters from Oidtmann et al. (2002). The zoospore production parameters were adapted from Makkonen et al. (2013) and Strand et al. (2012), while the zoospore survival and decay parameters were adapted from Oidtmann et al. (2002). We set the cannibalism rate to a value that limits the mean number of reproducing crayfish to 3200 individuals.

**TABLE 2:** The parameter values used in simulations.

Variable		Initial value/range	Unit
*P*	Oomycete zoospores	0	Individuals
*J*_1_	1‐year juveniles	0	Individuals
*J*_2_	2‐year juveniles	0	Individuals
*J*_3_	3‐year juveniles	0	Individuals
*S*	Adult crayfish	4000	Individuals
*M*	Infected juveniles	0	Individuals
*I*	Infected adults	10, 1, 1000	Individuals
*C*	Infected carcasses	0	Individuals
*a*	Zoospore production rate by I and M	200	day^−1^ Individuals^−1^
*d*	Zoospore production rate by C	1600	day^−1^ Individuals^−1^
*γ*_P_	Zoospore decay rate	0.1	day^−1^
*γ*_T_	Parasite decay rate in carcasses	0.01	day^−1^
*γ*_C_	Infected carcass decay rate	0.05	day^−1^
*r*_S_	Crayfish reproduction rate	100	year^−1^ Individuals^−1^
*μ*_J_	Juvenile mortality rate	0.004	day^−1^
*μ*_S_	Adult mortality rate	0.00082	day^−1^
*μ*_inf_	Infection mortality rate	0.1	day^−1^
*α*	Environmental transmission rate	10^−12^ … 10^−9^	day^−1^
*b*	Contact transmission rate	10^−9^ … 10^−5^	day^−1^
*c*	Cannibalism rate	5.5 × 10^−7^ … 2.2 × 10^−6^	day^−1^
*g*	Necrophagy transmission rate	5 × 10^−4^ … 7 × 10^−3^	day^−1^
*Δ*	Infection threshold	10^4^	Individuals
*Γ*	Variance of resistance	1 … 10^4^	

### 2.4 Simulation Settings

We used MATLAB R2022a (Mathworks) ode15s ODE solver and Matplotlib (Hunter 2007) for the simulations and figures, respectively. To find the steady state of the host population, the crayfish population was simulated for 1000 years using zeros for initial values of parasite spores (*P*) and infected crayfish (*M*, *I*, *C*). Nominal values of the infection threshold and the intraspecific necrophagy rate were *∆* = 1 × 10^−4^ and *g* = 5 × 10^−4^, respectively. The annual reproduction pulse prevented nontrivial equilibrium results, and the population remained in steady state indefinitely. Using the nominal cannibalism rate *c* = 1.1 × 10^−6^, the mean size of the parasite‐free crayfish population was approximately 3200 individuals, with periodic minima and maxima 2765 and 3729, respectively.

The simulation time for the parasite invasion dynamics was 50 years, and time step length for scoring the model dynamics was 1 day. The variables *∆* and *g* were varied to simulate different abiotic environments and oomycete strains. The crayfish population was first simulated for 30 years starting the crayfish population at 4000 adult individuals and again using zeros for initial values of *P*, *M*, *I*, and *C*. During that time, the population reached a discontinuous limit cycle, with the last years' population minima and maxima differing at most 3% from the parasite‐free steady state. Next, the simulation was continued by introducing 10 infected adult crayfish. The subsequent epidemiological dynamics were recorded for 20 years.

The crayfish population was considered extinct if the population size minimum dropped below one individual during the 20 years of epidemics. Respectively, the crayfish and the oomycete were considered coexisting if the minimum population size of both the crayfish and the oomycete zoospores was at least one individual through the whole simulation period.

## 3 Results

### 3.1 Survival of the Crayfish at Different Rates of Environmental and Contact Transmissions

**FIGURE 2:**
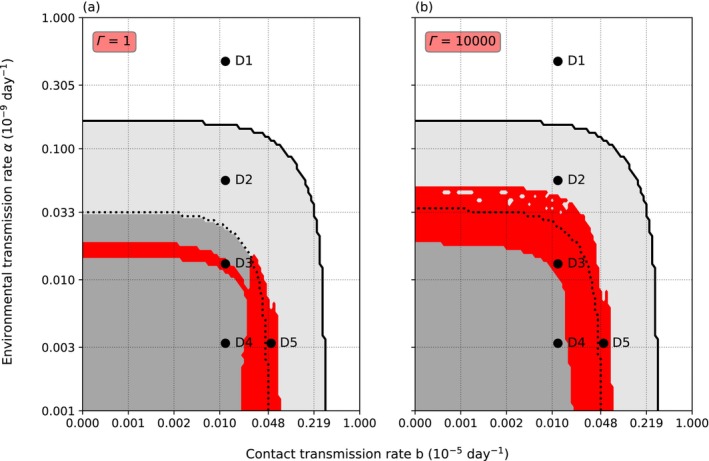
The survival of the crayfish and the oomycete. The figure shows the survival of the crayfish and the oomycete parasite using a 99 × 101 grid of contact transmission rate *b* (x‐axis) and environmental transmission rate *α* (y‐axis) 20 years after the introduction of the parasite. The gray area under the solid black line is the parameter area where the crayfish survives. Red color marks the survival of the oomycete. With high variance of host resistance *Γ* (b) the interaction of *b* and *α* can reduce the parasite population pointwise below one individual, which shows as gray spots on the red area. On the white area above the solid black line, both the crayfish and the oomycete disappear. In the area under the dotted line the crayfish population includes parasite‐free adult individuals continuously. To contrast, above the dotted line the crayfish population initially loses all adult members following the introduction of the oomycete. In this case, the crayfish population survives through the juvenile classes. (a) and (b) present the situations when the variance of resistance *Γ* = 1 and *Γ* = *∆* = 1 × 10^4^, respectively. Points D1–D5 mark parameter combinations that result in different dynamics and were explored further. (D1) *b* = 1.0 × 10^−7^, *α* = 4.6 × 10^−10^. (D2) *b* = 1.0 × 10^−7^, *α* = 5.7 × 10^−11^. (D3) *b* = 1.0 × 10^−7^, *α* = 1.3 × 10^−11^. (D4) *b* = 1.0 × 10^−7^, *α* = 3.3 × 10^−12^. (D5) *b* = 4.8 × 10^−7^, *α* = 3.3 × 10^−12^. In both subfigures, the infection threshold *∆* and cannibalistic necrophagy *g* rate have nominal values (*∆* = 1 × 10^4^, *g* = 5 × 10^−4^, respectively).

At nominal levels of infection, threshold *∆* and intraspecific necrophagy rate *g* (*∆* = 1 × 10^4^, *g* = 5 × 10^−4^) and low‐level variance of resistance (*Γ* = 1), mainly environmentally transmitted parasite could coexist with the host only within a narrow range of environmental transmission (*α*; Figure 2). Low *α* prevented the establishment of the parasite, while high *α* caused the extinction of both the crayfish and the oomycete. Similarly, coexistence with low or nonexistent *α* was possible only within a small range of *b*, which we call here simply contact transmission rate for brevity. Increasing the variance of resistance to *Γ* = *∆* widened the range of *α* yielding coexistence without affecting the range of suitable *b*. The crayfish became extinct at high values of *α* and *b*, independent of each other. However, high *b* lowered the extinction threshold value of *α*, and vice versa. Even if *α* and *b* remained under the extinction threshold, the crayfish population could lose all the adults: Surviving juveniles facilitated population recovery, but the population size did not reach original size within the 20 years since the first infection.

**FIGURE 3:**
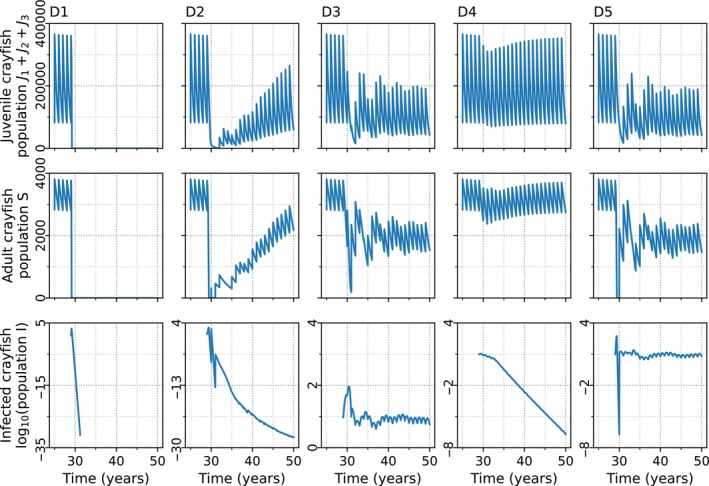
The time evolution of five basic dynamics when the level of individual variation of resistance is low (*Γ* = 1). The crayfish reproduce in discrete pulses and in steady state the population displays annual cycle. At the beginning of the cycle, the youngest juveniles (J1 in the model) comprise the majority of the combined juvenile classes shown at the top row. Following the parasite introduction after 30 years of simulation, the annual cycling continues, but the adult crayfish dynamics show five basic forms corresponding to the different parameter combinations (D1–D5). The initial data on the upper and middle row display the parasite‐free juvenile and adult crayfish populations, respectively. Because the number of infected adult crayfish during the coexistence remains low compared with the maximum peak, the bottom row shows the log_10_ value of the infected adult crayfish population. In all points except D5, contact transmission rate *b* = 1.0 × 10^−7^. Horizontal axis represents continuous time, and the discrete years of the reproductive bouts are marked on the continuous time axis. Vertical axis is the population size. (D1) When environmental transmission rate *α* = 4.6 × 10^−10^, the crayfish population falls to zero and does not recover. (D2) When *α* = 5.7 × 10^−11^, the number of adult crayfish falls initially to zero, but the crayfish start recovering. (D3) When *α* = 1.3 × 10^−11^, the crayfish and the oomycete coexist. (D4) When *α* = 3.3 × 10^−12^ the crayfish population first drops, but soon recovers. (D5) When *α* = 3.3 × 10^−12^ and *b* = 4.8 × 10^−7^ the parasite remains, the number of adult crayfish falls initially to zero, but the crayfish start to recover. Infection threshold *∆* and intraspecific necrophagy rate *g* have nominal values (*∆* = 1 × 10^4^, *g* = 5 × 10^−4^, respectively). The initial time marks the time of the first infection.

An introduction of the parasite into the crayfish population resulted in five basic population dynamical outcomes (Figure 3), corresponding to different combinations of *α* and *b*. The points D1–D5 (Figure 2) were chosen to represent the five distinct host dynamics using the nominal values (*∆* = 1 × 10^4^, *g* = 5 × 10^−4^). The dynamics at each point could vary according to the initial number of infected, the intraspecific necrophagy rate, and the variance of resistance. Point D1 lies within the extinction area, where high *α* causes an extinction of both the crayfish and the oomycete regardless of the value of *b*. Point D2 was selected from the area where the adults of the crayfish population disappeared completely soon after the parasite introduction, although the crayfish population as a whole ultimately survived through juvenile compensation and the oomycete disappeared. Point D3 (Figure 2) is located within the area where the combination of *α* and *b* lead to a coexistence of the crayfish and the parasite. Point D4 (Figure 2) is in the parameter area, where the crayfish population not only survives and the parasite perishes but also adult crayfish are constantly present. Finally, point D5 (Figure 2) is in the parameter area where the crayfish population survives in coexistence with the oomycete, but through the juvenile subpopulation. In all cases, the crayfish population declined at first, although when *α* was low, the decline could be small (D4). However, even at moderate levels of *α* or *b*, the number of uninfected adult crayfish could be close to zero for a short time, because the parasite reproduces efficiently in the infected hosts, resulting in high instantaneous zoospore densities. The number of zoospores in the system related to the five different dynamics of the crayfish population after the introduction of the oomycete. A coexistence of the host and the parasite resulted in a fluctuating presence of the zoospores in the water. The dense zoospore pressure that caused the extinction of both the crayfish and the oomycete resulted from the infection of the juvenile compartments in the population.

### 3.2 The Effect of the Initial Number of Infected Hosts

**FIGURE 4:**
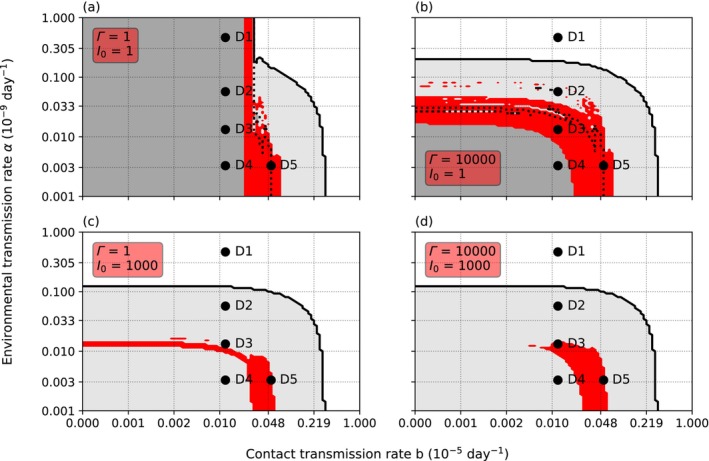
The effect of the initial number of infected crayfish. In subfigures a and c the variance of host resistance *Γ* = 1, while in b and d *Γ* is equal to the infection threshold *∆* (*Γ* = *∆* = 1 × 10^4^). The oomycete parasite coexists with the crayfish on the red area and is absent on the white and gray areas. When *Γ* = 1 and at the time of parasite introduction the initial number of infected crayfish *I*_0_ = 1 (a), the coexistence band is narrow, but reaches high values of environmental transmission rate *α*. The points D1–D4, which show different dynamics when *I*_0_ = 1000 (b), all have the same dynamics. Point D5 is now on the border of the area where the crayfish survive only through the decreased encounter rate between the juveniles and adults, i.e., the area between the solid and the dotted line. The area left to the solid black line shows the parameter area where the crayfish survives. Right to the solid black line both the crayfish and the oomycete disappear. To the contrary, when *Γ* = 1 × 10^4^ and *I*_0_ = 1 (b), the coexistence band is wide and reaches the area with low contact transmission rate *b*. When *Γ* = 1 and *I*_0_ = 1000 (c), the coexistence band is wide and reaches the area with low contact transmission rate *b*. The point D3 is not on the coexistence band, because the band runs at a lower value of *α*. When *Γ* = 1 × 10^4^ and *I*_0_ = 1000 (d), the coexistence area loses the band, but the unified area grows. The area under the solid black line shows the parameter area where the crayfish survives. Above the solid black line both the crayfish and the oomycete disappear. (D1) *b* = 1.0 × 10^−7^, *α* = 4.6 × 10^−10^. (D2) *b* = 1.0 × 10^−7^, *α* = 5.7 × 10^−11^. (D3) *b* = 1.0 × 10^−7^, *α* = 1.3 × 10^−11^. (D4) *b* = 1.0 × 10^−7^, *α* = 3.3 × 10^−12^. (D5) *b* = 4.8 × 10^−7^, *α* = 3.3 × 10^−12^. In all subfigures, infection threshold *∆* = 1 × 10^−4^, and the cannibalistic necrophagy rate *g* = 5 × 10^−4^.

**FIGURE 5:**
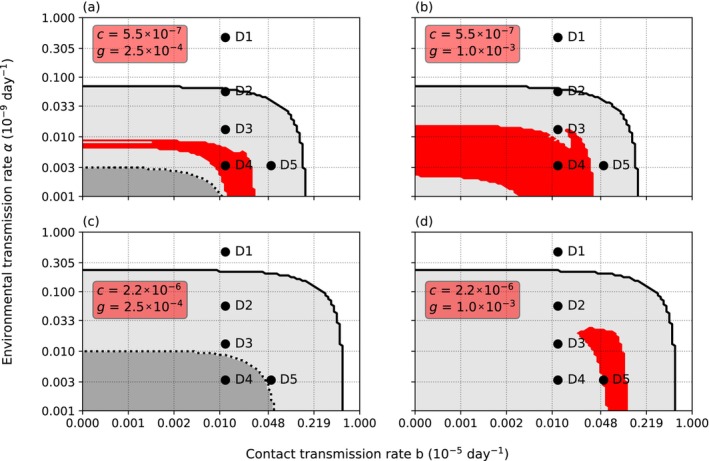
The effects of cannibalism rate *c* and intraspecific necrophagy rate *g*. On (a) and (b) cannibalism rate *c* is half from the nominal value *c* = 1.1 × 10^−6^ and on (c) and (d) the rate is double the nominal value. Similarly, on (a) and (c) the necrophagy rate *g* is half of the nominal value *g* = 5.0 × 10^−4^ and on (b) and (d) double the nominal value. At the higher cannibalism rate the parasite needs higher environmental transmission rate *α* or contact transmission rate *b* to invade. The light gray area represents the parameter area where adult crayfish are constantly present during the 20‐year period, whereas the dark gray area under the dotted line displays the parameter area where the adult hosts all initially die, but the crayfish population survives through the hiding juvenile class. Accordingly, high *c* decreases the possibility of coexistence (the red area) of the host and the parasite. To the contrary, high *g* increases the possible parameter area for coexistence but does not affect the overall survival of the host. In all subfigures the variance of resistance *Γ* = 10^4^ and the initial number of infected crayfish hosts at the time of the parasite introduction *I*_0_ = 1000.

The infection threshold (interpretable as parasite's infection capacity) affected the results. The effect was nonlinear and depended on the initial number of infected crayfish, *I*_0_. *Γ* affected the results at all initial values, but the effect was strongest, when *I*_0_ was low (Figures 4 and 5). With low *I*_0_ and low *Γ*, the parasite could establish only at a narrow range of *b*. However, a coexistence band similar to the one displayed when *I*_0_ = 10, appeared on the vertical axis (Figure 4a). Within this vertical band, the species coexisted regardless of the value of *α*. Increasing *Γ* limited the range on *α* but widened the range on *b* (Figure 4b), thus closely representing the situation with *I*_0_ = 10 (Figure 2b).

Increasing *I*_0_ to 1000 caused the initial disappearance of adult crayfish at all tested parameter ranges (Figure 4c,d). However, the 20‐year survival of the crayfish did not differ from the situation with a low initial value and high *Γ*. The separate smallest juvenile classes survived if *α* remained low. The coexistence of the crayfish and the parasite was possible at low values of *b* at low value of *Γ*, though the range of *α* was lower than when *I*_0_ was low (Figure 4c). Increasing *Γ* removed the horizontal coexistence band but widened the core coexistence area to include point D3 again (Figure 4d).

### 3.3 The Effects of Cannibalism, Intraspecific Necrophagy, and Variance of Resistance

Cannibalism *c* had a pronounced effect on the parasite's invasion success (Figure 5). High rate of *c* decreased the ranges of environmental transmission rate *α* and contact transmission rate *b* that allowed the establishment of the parasite. An increase in *c* decreased first the efficiency of *α*, thus increasing the importance of *b* as the transmission route. Accordingly, increasing *c* widened range of *α* and *b* leading to the extinction of the parasite, thus improving the survival of the host population. This was due to cannibalism's role as a population regulator. An increase in *c* decreases the density of the host population and the strength of density‐dependent environmental transmission. Thus, the improved survival of the population came at the cost of smaller population size. The effect was similar regardless of the initial number of infected crayfish hosts at the time of the parasite introduction.

**FIGURE 6:**
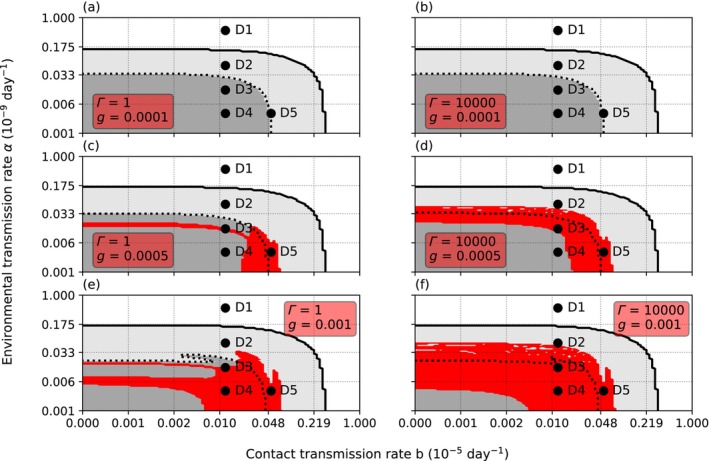
The effect of intraspecific necrophagy rate *g* at different rates of environmental (*α*) and contact transmissions (*b*). The subfigures a‐f show the effect of increasing the intraspecific necrophagy rate *g* at two different levels of variance of resistance (*Γ*) on the possibility of a coexistence of the crayfish and the oomycete. In (a) and (b), the necrophagy rate *g* is low and the parasite fails to persist. Above the solid black line both the crayfish and the oomycete disappear. Under the dotted line the crayfish population has parasite‐free adults through the whole time, whereas between the solid and the dotted lines initially all adult crayfish die, but the crayfish survive through the non‐infected juveniles. In (c) and (d), an increase in *g* makes the invasion of the parasite successful. The oomycete parasite coexists with the crayfish on the red area. In (e) and (f), further increase in *g* increases the parameter range suitable for an invasion, while simultaneously lowering the *α* threshold beyond which the crayfish survive only through the contacts of the juveniles with the adult. (D1) *b* = 1.0 × 10^−7^, *α* = 4.6 × 10^−10^. (D2) *b* = 1.0 × 10^−7^, *α* = 5.7 × 10^−11^. (D3) *b* = 1.0 × 10^−7^, *α* = 1.3 × 10^−11^. (D4) *b* = 1.0 × 10^−7^, *α* = 3.3 × 10^−12^. (D5) *b* = 4.8 × 10^−7^, *α* = 3.3 × 10^−12^. In all subfigures, *∆* = 1 × 10^4^.

Contrary to the cannibalism rate, the necrophagy rate *g* did not affect the overall survival of the crayfish host (Figure 5). However, the coexistence of the crayfish and the oomycete was dependent on *g* and the variance of resistance *Γ* (Figure 6). Low *g* made coexistence impossible, while increasing *g* increased the possibility of coexistence. Similarly, the range of coexistence increased with increasing *Γ* and high *g* allowed a constant adult crayfish presence regardless of *Γ*. Nevertheless, if *c* was kept constant, the change in *g* did not affect the survival of the crayfish during the 20 years, analogous to the effect of g when *c* was changed and *Γ* kept constant.

The susceptible adult class gets infected through environment, contacts with the still alive infected crayfish *M* and *I* and the infective carcasses *C*. Necrophagy decreases the number of infective carcasses but leads to a loop, where the production of zoospores decreases simultaneously with the increasing number of infected crayfish that finally succumb to the infection. Increasing *g* strengthens the loop, widening the ranges of *b* and *α* that cause the death of the adult crayfish class.

Regardless of *g*, *α* determines whether the zoospore density causes transmission to the small juvenile classes. Similarly, the contact rate *b* regulates the transmission between the juveniles regardless of *g*. From this follows that the value of *g* does not affect the extinction ranges of *b* and *α*.

## 4 Discussion

Crayfish plague not only provides a representative model for a detrimental disease with complex transmission but also is a considerable threat to numerous crayfishes outside North America. We have shown that a short‐term coexistence of the crayfish host and crayfish plague is possible in special circumstances after an initial invasion when variation in host resistance to environmental infections is present. Further, the variation in the host resistance affects the disease dynamics in an ecological timescale, which underlines the importance individual variation in affecting species‐level biodiversity and providing further material for selection to act toward more prolonged coexistence between the host and the disease. While our model showcased a highly virulent disease, *A. astaci* strains differ in virulence as well as crayfish populations in their resistance (Gruber et al. 2014; Makkonen et al. 2014; Martínéz‐Ríos et al. 2022). Thus, the introduction of a same species host with variance of resistance to an established host population through anthropogenic translocations could both increase and decrease the vulnerability of the population to emerging diseases on a case‐by‐case basis.

Understanding the role of variation in host resistance and the multitude of transmission modes of parasites is critical for disease control and management of many economically and ecologically important species (Murray 2009; Woolhouse 2019). Ecologically feasible models must capture the key host behaviors, life‐history specialties, and versatile transmission routes of parasites (Buhnerkempe et al. 2015; Valenzuela‐Sánchez et al. 2021). In our model, parasite transmission rate increased through contacts among the hosts, thus causing the extinction of the host population even when the transmission rate through the environment was generally low.

The results generally complied with the trade‐off model of parasite virulence (Anderson and May 1982; Alizon et al. 2009). In some cases, the host population not only survived but also the pathogen died out; at some parameter combinations, the host coexisted with the parasite. However, in most—if not all—of the empirical cases of European crayfish coexisting with *A. astaci* listed by Svoboda et al. (2017), the coexistence has involved latent carriers, that is, tolerant crayfish. While our model suggests that coexistence is possible without lowered virulence/increased resistance, the parasite tolerance as a separate mechanism should be addressed to fully understand the question of sustainable coexistence. Tolerance could decrease host mortality but increase parasite transmission rate (Miller, White, and Boots 2005).

Water is the most obvious transmission medium for *A. astaci*. Accordingly, research on crayfish plague has focused on the zoospore production (Oidtmann et al. 2002; Pavić et al. 2022; Strand et al. 2012) and on the environmental transmission with varying zoospore densities in the ambient water (e.g., Becking et al. 2015; Makkonen et al. 2014; Martínéz‐Ríos et al. 2022). The local zoospore density in the environment is partly dependent on external variables as the distribution of the infective stages depends on their sedimentation rate after active movement has ceased (Medema et al. 1998). Accordingly, flowing water has been shown to reduce transmission of aquatic parasites upstream (Bodensteiner et al. 2000; Radke, Ritchie, and Rowan 1961; Ray and Bartholomew 2013 but see Caprioli et al. 2013). From this follows that the transmission rate of aquatic parasites through contacts is difficult to quantify. The transmission through ambient water is always possible: from pure physical considerations, a contact between a parasite‐carrying and a parasite‐free individual can increase the zoospore density between the hosts to a much higher level than the dispersion into the water volume. Further motivation to consider physical contact comes from the weak chemotaxis of the *A. astaci* zoospores (Cerenius and Söderhäll 1984; Svensson 1978). Due to chemotaxis at scales relevant to infection, the zoospore density near the susceptible crayfish increases nonlinearly in the proximity of infected individuals. Thus, even when the transmission remains mechanically through the water, the rate of transmission can increase during contacts, cannibalism, and necrophagy. In this sense, the distinction between environmental and contact transmission is a continuous function of distance, and dichotomization is a pragmatic action. Ecologically importantly, the confrontational behavior of the host (Gruber et al. 2016; Roessink et al. 2022) increases the risk of contacts, while the strength of the effect may be affected by the structural complexity of the habitat (Corkum and Cronin 2004) and behavioral tendencies of individual hosts (Kortet, Hedrick, and Vainikka 2010). Also, shortage of resources could further increase aggression and as such contacts between individuals (Gruber et al. 2016; Roessink et al. 2022). Compared with simple density‐dependent transmission, the inclusion of contacts into the parasite transmission routes can change the transmission dynamics at varying host densities, and also independently of the variation in host resistance given the potentially overwhelmingly strong infection pressure.

The environmental transmission of crayfish plague depends on the infectivity of the parasite, that is, infection threshold of the *A. astaci* strain (Becking et al. 2015; Makkonen et al. 2014; Martínéz‐Ríos et al. 2022), which is likely subject to individual/strain‐specific variation like the host qualities (Makkonen et al. 2014). It could be argued that a large variance of resistance would increase the survival of the host population. Accordingly, our model projected that increasing variance in host resistance may increase the possibility of coexistence between the crayfish and the oomycete at low transmission rates, while rendering the host population susceptible to an invasion by a small initial number of the same parasites. Because the model does not include survival of the fittest, that is, evolutionary context providing potential rapid evolutionary increase in the average host resistance, the transmission rates and the virulence of the parasite remain constant. Thus, if the overall parasite transmission rate remains low, a high variation of resistance without selection allows the host and the parasite to coexist as populations even when an infection results in certain death of an average individual host.

Apart from the baseline environmental transmission rate and the increase in the rate due to the physical contacts, our model has two specific factors affecting the parasite transmission, namely host cannibalism and intraspecific necrophagy. Although the importance of these factors has not been quantified for *A. astaci*, we hypothesize that they could be important in outbreak scenarios. Cannibalism functions as a mechanism of population regulation (Abrahamsson 1966), and if the cannibalized conspecific is infected with the oomycete parasite, as an additional transmission route. However, the transmission through host cannibalism does not generally lead to catastrophic results or extinctions (Rudolf and Antonovics 2007; Koivu‐Jolma et al. 2023). According to our results, cannibalism could decrease the spread of an environmentally transmitted parasite by removing the infected hosts from the system before they reach the state of highest parasite zoospore production. Apart from the cannibalism, the crayfish also consume dead conspecific carcasses (Guan and Wiles 1998; Houghton, Wood, and Lambin 2017). For other microparasites, the act of eating parts of conspecifics provides an efficient transmission route for the parasite (Hamano et al. 2015; Imhoff et al. 2012; Schönherz et al. 2012). The transmission of *A. astaci* during necrophagy has not been studied. Regardless, the fundamental mechanism of increasing zoospore density during contact with an infected individual holds especially well during necrophagy. Rudolf and Antonovics (2007) concluded that neither cannibalism nor necrophagy are sufficient to cause an extinction of the host. Our model agrees with their results and presents some further insights into the dynamics. Although the necrophagy rate did not affect the 20‐year survival of the crayfish population, an increase in necrophagy increased the ranges of the pure environmental and the contact enhanced transmission rates that allowed the coexistence of the crayfish and the oomycete. Specifically, high rate of necrophagy caused an initial disappearance of adult crayfish class that was later compensated for by the surviving juveniles. This feature was based on the assumption that the smallest juveniles avoid contact with the larger conspecifics (Olsson and Nyström 2009) and thus do not normally encounter infected adult carcasses. However, the success of avoidance depends on the environmental complexity (Olsson and Nyström 2009) and the assumption might hold fully only in special circumstances. Notwithstanding, the result is comparable with earlier research, where age and/or space segregation has been shown to simultaneously uphold disease cycles and stabilize the dynamics (Tilman and Kareiva 1997, p.141–142, and the references therein). As the absolute rate of necrophagy, that is, functional response may depend on the availability of the carcasses (Knell, Begon, and Thompson 1996; Beasley, Olson, and DeVault 2012), the necrophagy rate is likely susceptible to environmental variation and population density. Thus, the environment‐specific decay rate of the infected carcasses used in the presented model should be tested for refined predictions.

Recent reports document that host life‐history characteristics in wild animals greatly influence host population responses to parasitism (Valenzuela‐Sánchez et al. 2021). Our results agree because dynamics were strongly coupled with the cyclic reproduction and age‐based behavioral segregation. Host life‐history affects the consumer–resource dynamics also in the scope of disease ecology (Valenzuela‐Sánchez et al. 2021). Our results complement the previous model that addressed crayfish plague dynamics but lacked the discrete time reproduction cycle (Koivu‐Jolma et al. 2023).

The presented model has components relevant also to other host–parasite systems. The disease dynamics bear close resemblance to the deadly African swine fever epidemics, including necrophagy and juvenile compensation (Gervasi and Guberti 2021; Pepin et al. 2020). On the other hand, the environment has been recognized as an important transmission route in traditionally density‐dependently modeled epidemics, for example, for the COVID‐19 virus (Vardoulakis et al. 2020). As such, our model represents a generalizable system with several factors affecting the parasite transmission.

In conclusion, our results indicate that during the initial wave of the epidemic, the combination of pulse‐formed reproduction and behavioral isolation of young juveniles from adults is enough to allow host parasite coexistence when the disease is highly virulent. This may have significant implications for managing populations that have faced outbreaks of emerging diseases and subsequent population crashes. Our model provides insights that are usable in the development of ecologically feasible epidemiological models for natural systems and in empirical studies resolving unknowns in disease ecology.

## Author Contributions

**Mikko Koivu‐Jolma:** conceptualization (equal), investigation (equal), visualization (lead), writing – original draft (equal), writing – review and editing (equal). **Raine Kortet:** conceptualization (equal), writing – review and editing (equal). **Anssi Vainikka:** conceptualization (equal), writing – review and editing (equal). **Veijo Kaitala:** conceptualization (equal), formal analysis (lead), investigation (equal), supervision (lead), visualization (supporting), writing – original draft (equal), writing – review and editing (equal).

## Conflicts of Interest

The authors declare no conflicts of interest.

## Data Availability

The codes used to generate the results of this study are openly available in Zenodo at https://doi.org/10.5281/zenodo.13789864.
